# Structure of a CGI-58 Motif Provides the Molecular Basis of Lipid Droplet Anchoring*

**DOI:** 10.1074/jbc.M115.682203

**Published:** 2015-09-08

**Authors:** Andras Boeszoermenyi, Harald Manuel Nagy, Haribabu Arthanari, Christoph Jens Pillip, Hanna Lindermuth, Rafael Eulogio Luna, Gerhard Wagner, Rudolf Zechner, Klaus Zangger, Monika Oberer

**Affiliations:** From the ‡ Institute of Molecular Biosciences, University of Graz, 8010 Graz, Austria,; the § Department of Biological Chemistry and Molecular Pharmacology, Harvard Medical School, Boston, Massachusetts 02115,; the ‖ Institute of Chemistry, University of Graz, 8010 Graz, Austria, and; the ¶ Institute of Biophysics, Medical University of Graz, 8010 Graz, Austria

**Keywords:** adipose triglyceride lipase (ATGL), lipid droplet, micelle, nuclear magnetic resonance (NMR), peptides, ABHD5, CGI-58, DPC, paramagnetic relaxation enhancement, peptide

## Abstract

Triacylglycerols (TGs) stored in lipid droplets (LDs) are hydrolyzed in a highly regulated metabolic process called lipolysis to free fatty acids that serve as energy substrates for β-oxidation, precursors for membrane lipids and signaling molecules. Comparative gene identification-58 (CGI-58) stimulates the enzymatic activity of adipose triglyceride lipase (ATGL), which catalyzes the hydrolysis of TGs to diacylglycerols and free fatty acids. In adipose tissue, protein-protein interactions between CGI-58 and the LD coating protein perilipin 1 restrain the ability of CGI-58 to activate ATGL under basal conditions. Phosphorylation of perilipin 1 disrupts these interactions and mobilizes CGI-58 for the activation of ATGL. We have previously demonstrated that the removal of a peptide at the N terminus (residues 10–31) of CGI-58 abrogates CGI-58 localization to LDs and CGI-58-mediated activation of ATGL. Here, we show that this tryptophan-rich N-terminal peptide serves as an independent LD anchor, with its three tryptophans serving as focal points of the left (harboring Trp^21^ and Trp^25^) and right (harboring Trp^29^) anchor arms. The solution state NMR structure of a peptide comprising the LD anchor bound to dodecylphosphocholine micelles as LD mimic reveals that the left arm forms a concise hydrophobic core comprising tryptophans Trp^21^ and Trp^25^ and two adjacent leucines. Trp^29^ serves as the core of a functionally independent anchor arm. Consequently, simultaneous tryptophan alanine permutations in both arms abolish localization and activity of CGI-58 as opposed to tryptophan substitutions that occur in only one arm.

## Teaser

**Background:** CGI-58 activates the key intracellular lipase ATGL.

**Results:** Solution structure of the N-terminal lipid droplet (LD)-binding motif of CGI-58 bound to dodecylphosphocholine micelles.

**Conclusion:** The LD-binding motif acts independently to anchor proteins to LDs and consists of two LD-binding arms.

**Significance:** The structure of the peptide LD anchor sheds light on the interaction of CGI-58 with LDs.

## Introduction

Triacylglycerols (TGs)^2^ are stored in lipid droplets (LDs) comprising a core of neutral lipids (TGs and sterol esters) surrounded by a monolayer of phospholipids (1). The protein “comparative gene identification 58 (CGI-58),” also known as α/β-hydrolase domain 5 (ABHD5), is an important stimulatory protein of the first step in intracellular lipolysis (2, 3). In this catabolic process, adipose triglyceride lipase (ATGL) catalyzes the hydrolysis of TGs stored in LDs to diacylglycerols and free fatty acids (FFAs). Hormone-sensitive lipase and monoacylglycerol lipase subsequently hydrolyze diacylglycerols and monoacylglycerols, respectively, to generate FFAs and glycerol molecules (4).

Mutations in the human gene encoding CGI-58 lead to neutral lipid storage disease with a severe skin defect termed ichthyosis (NLSD-I) (5). Although the ATGL stimulating function of CGI-58 appears causative for the neutral lipid storage phenotype in affected patients, the frequently observed symptoms of hepatomegaly, hepatic steatosis, and ichthyosis are indicative of an ATGL-independent function of CGI-58 (6–8).

The rate of intracellular lipolysis on the surface of LDs depends on post-translational modification events, multiple protein-protein interactions and lipase-ligand interactions at the lipid-water interphase (2, 9–23). Under basal conditions, CGI-58 binds to the LD coating protein perilipin 1 in 3T3-L1 adipocytes (20). In this state, CGI-58 does not interact with ATGL and ATGL activity remains low (24, 25). Phosphorylation of perilipin 1 and CGI-58 by protein kinase A (PKA) leads to rapid release of CGI-58 from perilipin 1-coated LDs and subsequent activation of ATGL (18, 26).

The role of perilipin 1 in the recruitment of CGI-58 to LDs is not well understood. CGI-58 has been shown to activate ATGL also on artificial LD substrates lacking perilipins (12, 15). Currently, it remains unknown whether CGI-58-mediated activation of lipolysis occurs due to increased access to the substrate, conformational changes induced in ATGL, increased product release (*e.g.* channeling the produced FFA away from the reaction site), or increased lipolysis due to interaction with fatty acid-binding proteins (16). Interestingly, the selectivity of ATGL hydrolysis at the *sn*-2 position of the glycerol backbone broadens to the *sn*-1 position upon interaction with CGI-58 (27). The lack of high-resolution structures of CGI-58 and ATGL or the protein-protein complex in the presence of a LD surface represents a major bottleneck in understanding the function of these proteins and their interaction surfaces.

The interaction of CGI-58 with ATGL occurs within the N-terminal patatin domain-related region of ATGL (12, 28). A homology model of CGI-58 reveals a core α/β-hydrolase structure consisting of eight mostly parallel β-strands surrounded by α-helices and loops, a cap region comprising α-helices, and a short mostly unstructured N-terminal tryptophan-rich stretch (15). Almost the entire CGI-58 protein is required to activate ATGL, because CGI-58 variants with major deletions from the N or C terminus are not capable of activating ATGL (15). The N-terminal Trp-rich region serves an essential role in the localization of CGI-58 to LDs, which remains a strict requirement for ATGL activation (15).

To better understand the mechanism of CGI-58 LD binding, we solved the structure of the N-terminal fragment of CGI-58 (peptide Val^10^ to Lys^43^, CGI-58_V10-K43) bound to dodecylphosphocholine (DPC) micelles, which serve as excellent mimics of the LD surface. The structure reveals that the region Ser^19^-Cys^30^ constitutes a LD anchor motif with Trp^21^ and Trp^25^ forming a hydrophobic core along with the hydrophobic residues Leu^22^ and Leu^26^. This hydrophobic core constitutes the left arm of the anchor. The more isolated Trp^29^ is flanked by two prolines (Pro^27^ and Pro^31^) and serves as the functionally independent right arm of the anchor.

A fusion protein containing just the CGI-58 LD anchor motif (amino acids 19–35) fused to yellow fluorescent protein (YFP) localizes to LDs supporting the concept of the LD-anchor as an independent functional motif. Selective permutations converting single tryptophans of the LD anchor to alanines do not alleviate the ability of CGI-58 to localize to LDs or activate ATGL. However, substitutions in both arms of the LD anchor (W21A and W29A) abolish the ability of CGI-58 to localize to LDs and to activate ATGL.

## Experimental Procedures

### 

#### 

##### Generation of Trp Variants of CGI-58

Wild type (WT) and all mutants of CGI-58 were cloned into the plasmid pEYFP-N1 (BD-Biosciences Clontech) coding for a C-terminal EYFP tag. Generation of wild type-CGI-58 (WT-CGI-58) and the point-mutated variants W21A, W29A, and W21A/W25A was described earlier (15). The single point mutant W25A and the double mutant W21A/W29A were generated by site-directed mutagenesis of a vector encoding for WT-CGI-58 with a C-terminal EYFP tag. The N-terminal peptide containing just the LD anchor mCGI_19–35 was generated upon amplification of the sequence using the forward primer mCGI_19 YFP N1 forward, 5′-GTGATGACCTCGAGATGTCAGGATGGCTG-3′; and the reverse primer mCGI_35 YFP N1 reverse, 5′-GGAATAGGATCCGCTGATGTAGATGTGGGACACC-3′ followed by ligation into XhoI and BamHI sites of the vector. The correctness of all sequences was verified by DNA sequencing (LGC Genomics, Berlin, Germany).

##### Cellular Localization of mCGI-58 Variants

For localization studies, monkey embryonic kidney cells (COS-7, ATCC CRL-1651) were transfected with expression vectors (pEYFP-N1) encoding WT full-length and point mutants of mouse CGI-58 (mCGI-58) with a C-terminal fusion of YFP. COS-7 cells were maintained in Dulbecco's modified Eagle's medium (DMEM, Life Technologies) containing 4.5 g/liter of glucose, 10% fetal calf serum (FCS), and penicillin/streptomycin under a humidified atmosphere, 37 °C, and 5% CO_2_. COS-7 cells were seeded on glass coverslips in 6-well plates (1.2 × 10^5^ cells/well) and transfected with YFP-tagged full-length or mutated mCGI-58 variants. 24 h after transfection, cells were incubated for 20 h in DMEM containing FCS, and supplemented with oleic acid (400 μm) complexed to fatty acid-free BSA (Sigma) in a ratio of 3:1 to increase LD formation. LDs were stained with HCS LipidTOX Red Neutral Lipid stain (Life Technologies) and incubated for 10 min at 37 °C. Microscopy was performed using a Leica TCS SP5 confocal microscope (Leica Microsystems GmbH) with a HCX PL APO CS 63× 1.2 water objective. YFP fluorescence was excited at 514 nm and detected at 522–558 nm. LipidTOX was excited at 633 nm and detected at 650–669 nm. Transmission images of cultured cells were also recorded. All presented experiments were repeated independently at least three times.

##### Preparation of Cell Extracts for Triglyceride Hydrolase Assay

COS-7 cells were transiently transfected with the different CGI-58 clones and pcDNA4/HisMax coding for His-tagged ATGL (28) with Metafectene^TM^ (Biontex GmbH) as described earlier (29). The cells were disrupted by sonication and resuspended in lysis buffer (0.25 m sucrose, 1 mm dithiothreitol, 1 mm EDTA, 20 μg/ml of leupeptine, 2 μg/ml of antipain, 1 μg/ml of pepstatin, pH 7.0). Then, nuclei and unbroken cells were removed by centrifugation at 1000 × *g* at 4 °C for 5 min, and the supernatants were used for triglyceride hydrolase activity assays.

##### Assay for Triglyceride Hydrolase Activity

The substrate for the triglyceride hydrolase activity assay was prepared as described previously with minor modifications (29). Briefly, triolein and [9,10-^3^H]triolein (10 μCi/ml) were emulsified in the presence of phosphatidylcholine/phosphatidylinositol using a sonicator (Virsonic 475, Virtis, Gardiner, NJ) and adjusted to 2.5% BSA (FFA free). The final substrate concentration was 0.3 μmol/ml of triolein and 0.15 mg/ml of phosphatidylcholine/phosphatidylinositol (3:1). The reaction mixture was prepared of lysates containing overexpressed HisMax-mATGL (30 μg total protein) and the lysates expressing the different variants of CGI-58 (30 μg of total protein). Activity assays were performed using 0.1 ml of cell lysate mixture and 0.1 ml of substrate in a water bath at 37 °C for 60 min. The reaction was terminated by adding 3.25 ml of methanol/chloroform/heptane (10:9:7) and 1 ml of 0.1 m potassium carbonate, 0.1 m boric acid, pH 10.5. After centrifugation at 800 × *g* for 20 min, the radioactivity in 0.2 ml of the upper phase was determined by liquid scintillation counting.

##### Statistical Analysis

TG hydrolase activity measurements were performed in triplicates. Measured activities are represented as mean ± S.D. Statistical significance was determined by the Student's unpaired two-tailed *t* test. Groups were considered to be significantly different for *p* < 0.05 (*), *p* < 0.01 (**), and *p* < 0.001 (***).

##### Cloning and Expression of His_6_-smt3-TEV-mCGI-58_V10-K43

The coding sequence of mouse CGI-58 (mCGI-58) was available in a pSumo vector as described (5). An N-terminally truncated mCGI-58 variant, starting with the nucleotides coding for Val^10^, was subcloned into a modified pSumo vector carrying a tobacco etch virus (TEV) protease cleavage site. The oligonucleotides 5′-GTAGACTTGGATCCGTGGACTCGGCAGACG-3′ and 5′-GGAACCCTCGAGTCATCAGTCTACTGTGTGGC-3′ were used as forward and reverse primers, respectively. Then a stop codon was inserted by site-directed mutagenesis to truncate mCGI-58 after Lys^43^ and produce the His_6_-smt3-TEV-mCGI-58_V10-K43 construct. This vector was transformed into BL21(DE3) *Escherichia coli* cells and cultures were grown in Luria broth (Miller, EMD Millipore Corp., Billerica, MA) medium containing 40 mg/liter of kanamycin up to an *A*_600_ of 1.0 before induction with 0.5 mm isopropyl β-d-1-thiogalactopyranoside. After 3 h expression at 37 °C the cells were harvested by centrifugation for 20 min at 4 °C and 3,500 × *g*. ^15^N- and ^13^C-labeled His_6_-smt3-TEV-mCGI-58_V10-K43 was expressed in minimal medium containing 1 g/liter of [^15^N]NH_4_Cl and 2 g/liter of [^13^C]glucose.

##### Purification of His_6_-smt3-TEV-mCGI-58_V10-K43

The cell pellet from a 1-liter culture was resuspended in 50 ml of buffer 1 (20 mm Tris-HCl, pH 7.8, 500 mm NaCl, 30 mm imidazole, 1% Nonidet P-40, 3.5 mm β-mercaptoethanol, 1 tablet of Roche EDTA-free protease inhibitor, 1 mg/ml of lysozyme, and 750 units of benzonase* nuclease HC (purity >90%, Novagen)). The cells were lysed by sonication and the soluble fraction was separated by centrifugation at 39,000 × *g* for 40 min at 4 °C. This fraction was incubated with 4 ml of Ni-NTA beads (Qiagen), pre-equilibrated in buffer 2 (20 mm Tris-HCl, pH 8.0, 350 mm NaCl, 10 mm imidazole, 3.5 mm β-mercaptoethanol), for 60–90 min at 4 °C on a nutator. The Ni-NTA beads were washed with 50 ml of buffer 2, buffer 3 (20 mm Tris-HCl, pH 8.0, 1000 mm NaCl, 10 mm imidazole, 3.5 mm β-mercaptoethanol), and buffer 4 (20 mm Tris-HCl, pH 8.0, 350 mm NaCl, 40 mm imidazole, 3.5 mm β-mercaptoethanol), respectively, to remove nonspecifically bound proteins. His_6_-smt3-TEV-mCGI-58_V10-K43 was eluted with 30 ml of buffer 5 (20 mm Tris-HCl, pH 8.0, 350 mm NaCl, 250 mm imidazole, 3.5 β-mercaptoethanol) and concentrated with a 3-kDa cut off centrifugal filter unit (Amicon Ultra-15, Millipore) after addition of 10 mg of deuterated dodecylphosphocholine (DPC-*d*_38_, Cambridge Isotope Laboratories). The buffer was exchanged to buffer 6 (15 mm Na_2_HPO_4_, 5 mm KH_2_PO_4_, 300 mm NaCl, 1 mm EDTA, 1 mm DTT, pH 6.8) in the filter device and another 10 mg of DPC-*d*_38_ were added after concentration to ∼1 ml. The His_6_-smt3 fusion tag was cleaved off with TEV protease and the cleaved sample was diluted to 10 ml in buffer 6. The solution was subsequently applied onto a 5-ml pre-packed Ni-NTA column (GE Healthcare, pre-equilibrated in buffer 6). Approximately 15 ml of flow through containing pure peptide V10-K43 were collected, concentrated, and exchanged to buffer 7 (17.6 mm NaH_2_PO_4_, 2.4 mm Na_2_HPO_4,_ 50 mm NaCl, 1 mm EDTA, 5 mm DTT, pH 6.0). For NMR experiments, 5% D_2_O were added to a 1 mm sample of peptide V10-K43 (within this text, this peptide is referred to as peptide “V10-K43”).

##### Preparation of the Synthetic Peptide “G18-E39”

A 22-residue peptide containing the amino acid sequence GSGWLTGWLPTWCPTSTSHLKE corresponding to residues Gly^18^ to Glu^39^ of mCGI-58 (referred to as peptide “G18-E39”) was purchased from a commercial supplier (Peptide Special Laboratories, Heidelberg). For NMR experiments, it was dissolved in buffer 7 containing 100 mm DPC-*d*_38_ at a concentration of 1 mm and measured upon addition of 5% D_2_O.

##### NMR Experiments with Peptides V10-K43 and G18-E39

All experiments on peptides V10-K43 and G18-E39 were recorded in the presence of DPC micelles. Due to the low solubility of the peptides in aqueous solvents, assignment of CGI-58 peptides V10-K43 and G18-E39 in the absence of detergent was not feasible.

Standard backbone experiments (30) (HNCA, HN(CA)CO, HNCACB) of the peptide V10-K43 were recorded on a 600 MHz Bruker spectrometer equipped with an Avance I console and a cryogenically cooled TCI 5-mm probe. The side chain experiments HCCH-TOCSY and (H)C(C-CO)NH-TOCSY were recorded on a 500 MHz Varian spectrometer equipped with a Unity Inova console and an HCN cryoprobe. An H(CC-CO)NH-TOCSY was recorded on a 750 MHz Bruker spectrometer equipped with a cryogenically cooled TCI 5-mm probe and an Avance III console. ^15^N- and ^13^C-dispersed three-dimensional NOESY experiments were recorded on a 700 MHz Varian spectrometer equipped with an Agilent dd2 console and HCN salt-tolerant cryoprobe (150 ms mixing time) and a 900 MHz Bruker spectrometer with Avance II console and TCI cryoprobe (80 ms mixing time), respectively. All backbone and side chain experiments, with the exception of the (H)C(C-CO)NH-TOCSY, were recorded using non-uniform sampling where 15–20% of the indirect dimension grid was sampled using Poisson Gap Sampling (31). Heteronuclear three-dimensional NMR experiments were recorded at 310 K to minimize transversal relaxation times on residues immersed in the micelles. Concomitantly, this temperature reduced the dynamic range of the sample, as residues exposed to the solvent exchange more rapidly with water at elevated temperatures and therefore lost some of their otherwise high signal intensity. Homonuclear two-dimensional TOCSY and two-dimensional NOESY spectra of peptide G18-E39 were recorded on a 900 MHz Avance II Bruker spectrometer equipped with a cryogenically cooled probe at 303 K using 90 and 200 ms mixing times, respectively. Uniformly collected NMR spectra were processed with NMRpipe (32). Non-uniformly sampled spectra were processed with hmsIST in combination with NMRpipe (31, 33). All NMR spectra were visualized and analyzed with CcpNmr (34).

Relaxation experiments were recorded on the peptide V10-K43 at 310 K. Longitudinal (*T*_1_) relaxation times were measured on a 600 MHz Avance I Bruker spectrometer equipped with a cryogenically cooled probe as a pseduo three-dimensional data set. Relaxation delays for *T*_1_ were 10, 50, 100, 150, 200, 300, 500, 800, 1000, 1200, 1500, and 1800 ms, respectively. Spin-spin (*T*_2_) relaxation times and heteronuclear NOE (^15^N[^1^H] NOE) experiments were recorded on an 800 MHz Bruker spectrometer equipped with an Avance III console and a room temperature probe. The relaxation delays used in the *T*_2_ data series were 20, 40, 60, 80, 100, 120, 140, and 160 ms, respectively. The interleaved ^15^N[^1^H] NOE experiment was recorded with a 2-s saturation delay. Peak heights were integrated with CcpNmr and *T*_1_ and *T*_2_ times were also calculated within the CcpNmr software suit. ^15^N[^1^H] NOE peak heights were evaluated with relax (35, 36).

^15^N and ^13^C paramagnetic relaxation enhancements of V10-K43 were obtained from *T*_1_ delay modulated ^1^H-^15^N HSQC and ^1^H-^13^C HSQC spectra, respectively, at 303 K. In both the above mentioned series of experiments, samples of V10-K43 were titrated with 0, 2, 4, 6, 8, and 10 mm gadolinium-diethylenetriamine pentaacetic acid-bismethylamide (Gd(DTPA-BMA)) and relaxation delays were 70, 150, 250, 350, 500, 750, 1000, 2000, 3000, and 5000 ms, respectively. Using the program relax, peak intensities were fitted as described (37) to obtain *T*_1_ relaxation times. The spectra were recorded on an 800 MHz Bruker spectrometer equipped with an Avance III console and a TCI 5-mm cryogenically cooled probe.

Paramagnetic relaxation enhancement (PRE) values were extracted and converted to distance restraints according to published protocols (37, 38). We calculated the hydrodynamic radius of the DPC micelles to be ∼30 Å (*r* = 30 Å) based on the translational diffusion coefficient measured by dynamic light scattering (described below). The constants *g* (7.98 Å) and *k* (253 mm^−1^ Å^3^) were used according to the literature (38). Gd(DTPA-BMA) was purchased as Gadodiamide from (Toronto Research Chemicals, Toronto, Canada) and added from a 60 mm stock in H_2_O. PRE-derived distance restraints were weighted at 30% with respect to NOEs, upper and lower boundaries of ±2 Å were used.

NOEs from ^15^N- and ^13^C-dispersed NOESY-HSQCs of V10-K43 and a homonuclear NOESY of peptide G18-E39 were picked, assigned, integrated, and converted to distance restraints in CcpNmr (34). Restraints for torsion angles were prepared with TALOS+ (39) and PRE-derived distance restraints were calculated as described above. 100 structures were calculated with a simulated annealing protocol using CYANA (40) and the 20 structures with the lowest energy target functions were chosen for deposition. Structures were visualized with PyMOL (41) and the quality of the structures was assessed with PSVS (42) and iCING (43).

##### Circular Dichroism (CD) Spectroscopy

For CD spectroscopy, a sample of peptide V10-K43 was prepared at 0.76 mg/ml in buffer 7 and DPC as described above for the preparation of NMR samples. A corresponding baseline sample was prepared without peptide. Data were measured with a Jasco J-715 spectropolarimeter at 0.01-cm path length between 190 and 260 nm with 0.1-nm steps, 1-nm bandwidth, and 1-s averaging time at 50 nm min^−1^ scanning speed. 10 spectra were recorded, averaged, and baseline corrected.

##### Dynamic Light Scattering

The hydrodynamic radius of micelles in the presence of peptide was measured to be 30 Å with dynamic light scattering (Protein Solutions DynaPro MS/X instrument, Protein Solutions Inc., Lakewood, NJ). The dynamic light scattering micelles with the peptides were measured at 5-s acquisition time, 30% laser power, and 20 acquisitions. As a reference, 100 mm DPC was measured in H_2_O. The measured radius of 21 Å for the free micelle is in good agreement with the literature (38, 44, 45). The difference in micelle size is presumably due to higher salt and DPC concentration, which is a direct result of the peptide preparation process using spin concentrators.

##### Protein Data Bank (PDB) and Biological Magnetic Resonance Bank (BMRB) Accession Numbers

Coordinates and NMR resonance assignments have been deposited in the Protein Data Bank (PDB code 5A4H) (46) and Biological Magnetic Resonance Data Bank (BMRB code 25684) (47).

## Results

### 

#### 

##### The LD-binding Motif of CGI-58 Tolerates the Loss of Any Single Tryptophan Residue, but Not the Simultaneous Loss of Trp^21^ and Trp^29^

**FIGURE 1.**
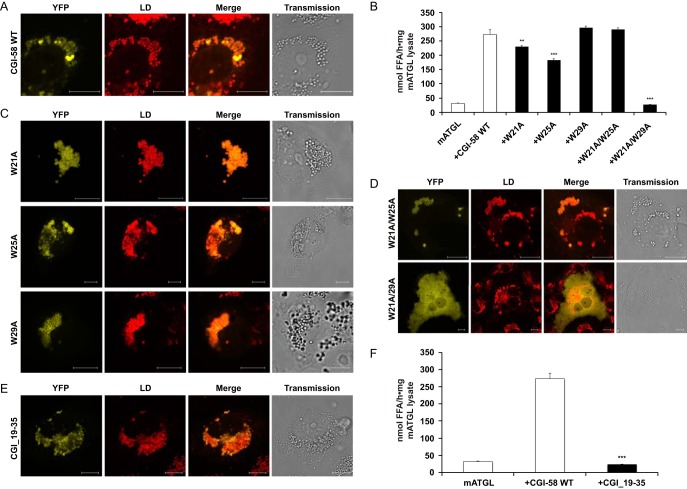
**The N-terminal region of CGI-58 is a fully functional LD anchor.** Substitution of its two terminal tryptophans abrogates the ability of CGI-58 to localize to LDs and to activate ATGL. *A*, confocal laser-scanning microscopy image of YFP tagged wild type (WT) CGI-58 expressed in oleate laden *COS-7* cells (*first column*) co-localizes with LDs. HCS LipidTOX-stained LDs (*second column*) are overlaid with YFP-CGI-58 in *third column*. The *fourth column* represents the transmission image of the respective cells. *Scale bars*: 10 μm. *B,* testing the ability of CGI-58 and several CGI-58 variants to activate ATGL. All single Trp variants (W21A, W25A, and W29A) activate ATGL. The double variant W21A/W25A retains the full ability to activate ATGL, but W21A/W29A cannot activate ATGL. Presented data are one representative from three independent experiments, mean ± S.D. *C,* single Trp variants of CGI-58 co-localize with LDs. *D,* the double variant W21A/W25A co-localizes with LDs, but the double variant W21A/W29A does not. *E,* YFP-tagged CGI-58 peptide ranging from Ser^19^ to Ser^35^ (CGI_19–35) co-localizes to LDs. *F,* CGI_19–35 does not activate ATGL.

Full-length mammalian CGI-58 localizes to LDs in differentiated 3T3-L1 adipocytes and *COS-7* cells (Fig. 1*A*). This interaction involves the tryptophan-rich N terminus of CGI-58 (Trp^21^, Trp^25^, and Trp^29^) in LD binding (15, 20, 48, 49). CGI-58 lacking the first 31 residues or harboring changes in the three N-terminal Trp residues failed to co-localize to LDs (15), whereas conversion of Trp^21^ to alanine (W21A) alone did not prevent the localization of CGI-58 to LDs or the activation of ATGL (Fig. 1*C*). Similarly, CGI-58 variants W25A and W29A retained their ability to localize to LDs; although somewhat reduced ATGL stimulation was observed for the variants W21A and W25A (Fig. 1, *B* and *C*).

Next, we generated variants with double amino acid exchanges, W21A/W25A, and W21A/W29A. Although the W21A/W25A variant localized to LDs and concomitantly activated ATGL with undiminished capacity the W21A/W29A variant failed to localize to LDs and to activate ATGL (Fig. 1, *B* and *D*). This strengthens the functional relevance of proper CGI-58 localization observed previously (15) and supports the proposed prominent role for the N-terminal region of CGI-58.

To investigate whether the N-terminal region self-sufficiently localizes to LDs, we expressed a YFP-tagged peptide ranging from Ser^19^ to Ser^35^ (CGI_19–35) in *COS-7* cells and monitored its intracellular localization. The peptide localized to LDs in a manner reminiscent of wild type CGI-58 (Fig. 1, *A* and *E*). However, when we tested the peptide CGI_19–35 for its ability to activate the triacylglycerol (TG) hydrolase activity of ATGL, we did not observe any stimulating effect (Fig. 1*F*).

##### Resonance Assignments of the CGI-58 Peptides G18-E39 and V10-K43

**FIGURE 2.**
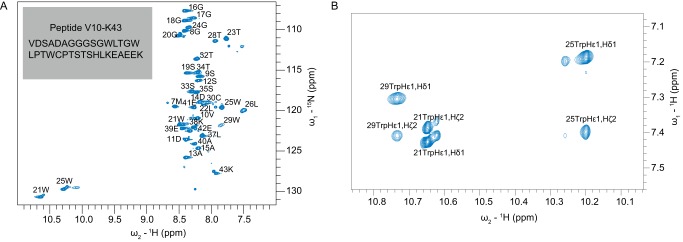
**Assignment of the peptides V10-K43 and G18-E39.** *A,* ^1^H-^15^N HSQC spectrum of the V10-K43 peptide bound to DPC micelles, with backbone NH and tryptophan side chain NHϵ assignments. *B*, aromatic region of the homonuclear NOESY spectrum of the G18-E39 peptide, also bound to DPC micelles.

To characterize the three-dimensional structure of the N-terminal LD binding region, we determined the solution structure using NMR spectroscopy. 96% of backbone and 74% of side chain resonances of the peptide V10-K43 bound to DPC micelles were assigned (Fig. 2*A*). The heteronuclear ^15^N- and ^13^C-dispersed NOESY-HSQC spectra of the peptide V10-K43 did not contain a sufficient number of cross-peaks for structure calculation, which is likely attributed to dynamics of the sample at 310 K. Therefore, we recorded homonuclear TOCSY and NOESY experiments on the synthetic 22-residue peptide mCGI-58_G18-E39 (G18-E39) at a lower temperature of 303 K. The resonance assignments from the longer peptide V10-K43 could be transferred and consequently, we assigned all NH and Hα resonances of the unlabeled 22-residue peptide G18-E39 with the exceptions of the His^36^-Hα proton and resonances corresponding to Gly^18^ at the N terminus. 82% of non-water exchangeable side chain protons were also assigned. Trp^21^ and Trp^25^ Hϵ-1 and Nϵ-1 resonances were assigned with heteronuclear ^1^H-^15^N HSQC and ^15^N-dispersed NOESY-HSQC spectra. The Trp^29^ Hϵ-1 resonance and additional Trp aromatic side chain resonances were assigned from the homonuclear spectra (Fig. 2*B*). Assignments were deposited in the BMRB accession number 25684.

##### The N-terminal Peptide of CGI-58 Reveals a Mostly Unstructured Anchor

**FIGURE 3.**
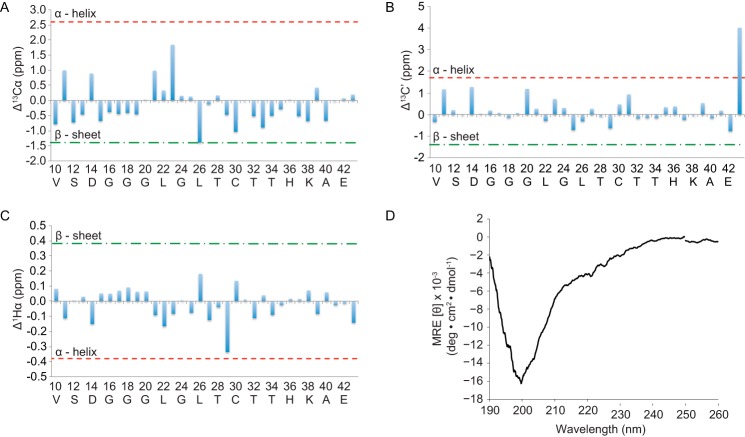
**The V10-K43 peptide adopts a random coil conformation.** Downfield and upfield shifts of ^13^Cα, ^13^C′, and ^1^Hα resonances are sensitive probes for secondary structure elements when compared with averaged random coil values. *Panels A–C* show ^13^Cα (*A*), ^13^C′ (*B*), and ^1^Hα (*C*) chemical shift deviations from random coil values for resonances of the peptide V10-K43. *Bars* represent individual residues. Deviations indicative of an α-helix (*red*) or a β-sheet (*green*) are marked by *dotted lines. D*, the circular dichroism spectrum of V10-K43 with a minimum at 200 nm reaffirms the unstructured nature of the peptide. *MRE*, mean residue ellipticity.

The observed chemical shifts of a protein or peptide are sensitive indicators of α-helix and β-sheet elements when compared with average random coil shifts. Thus, the assignments of the N-terminal peptides of CGI-58 reveal initial per-residue information on secondary structure elements. In particular, downfield shifts of ^13^Cα and ^13^CO and upfield shifts of ^1^Hα resonances with averaged changes of 2.6, 1.7, and 0.38 ppm, respectively, would indicate an α-helix. Upfield shifts with averaged changes of 1.4 ppm for ^13^Cα and ^13^CO, and downfield shifts of 0.38 ppm for ^1^Hα, would indicate β-sheets (50–52). Examination of ^13^Cα, ^1^Hα, and ^13^CO shifts of the CGI-58 peptide V10-K43 in DPC micelles did not provide an indication of α-helix or β-sheet elements, although a propensity for helix formation might be inferred for the region Gly^20^-Gly^24^ (Fig. 3, *A–C*). Additionally, a circular dichroism (CD) spectrum of the peptide V10-K43 showed characteristics of a mostly unfolded peptide with a minimum around 200 nm (Fig. 3*D*). These experimental results are in good agreement with structure predictions. Secondary structure predictions indicate peptide V10-K43 to be partially unstructured, with a propensity to form α-helices (PSIPRED, Jpred (53, 54)). A homology model of CGI-58 based on the structure of the *Aspergillus niger* epoxide hydrolase also indicates helical and unstructured parts in the N terminus of CGI-58 (15).

##### Relaxation Analysis of the CGI-58 Peptide V10-K43 Confirms Binding to DPC Micelles and Independent Motion of Three Different Segments within the Peptide

To investigate the dynamic behavior of the individual residues of the CGI-58 peptide V10-K43, we recorded longitudinal (spin-lattice, *T*_1_) and transversal (spin-spin, *T*_2_) relaxation experiments and a set of heteronuclear NOE (^15^N[^1^H] NOE) experiments. *T*_1_ times increase when the tumbling rate of a residue slows down, whereas a decrease in the spin-spin relaxation (*T*_2_) time corresponds to a decrease of tumbling rate and increase in protein size. The ^15^N[^1^H] NOE experiment measures the change in steady-state populations of ^15^N spins when the attached proton spins are saturated. This experiment is specifically sensitive to changes of the correlation time for internal motion and reflects variations in protein backbone dynamics on the pico- to nanosecond time scale. Reduced and negative NOEs correspond to rapid internal motion, whereas ratios close to 0.8 indicate more stable segments of proteins and peptides (55).

**FIGURE 4.**
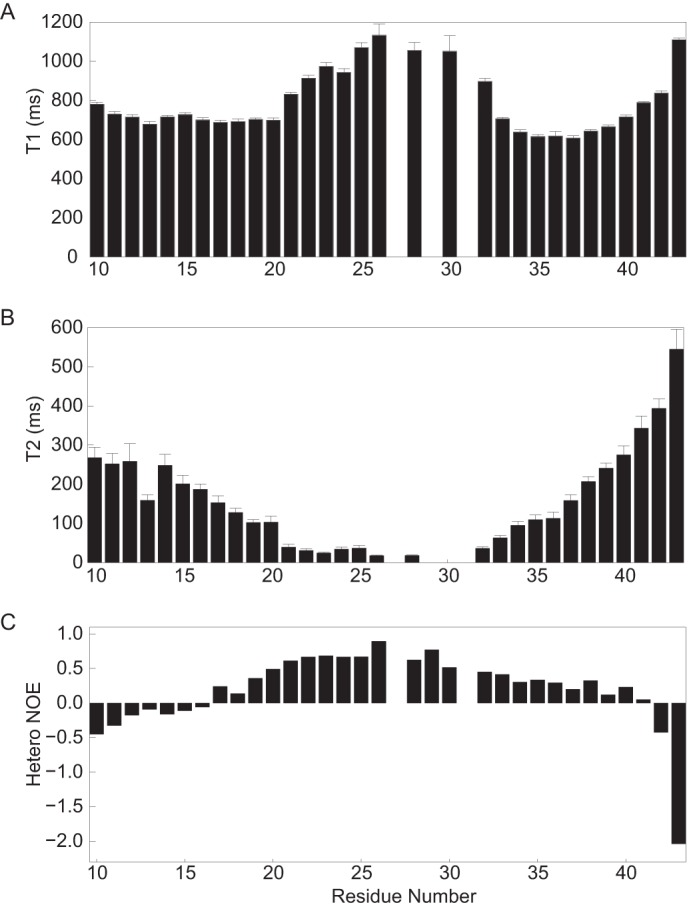
**The V10-K43 peptide comprises three independently moving regions.** Motion dynamics of the LD anchor Ser^19^-Cys^30^ mimic those of a large protein, whereas the flanking regions fluctuate as independently moving peptides. *A,* longitudinal relaxation times (*T*_1_); *B,* transversal relaxation times (*T*_2_); *C,* heteronuclear ^15^N[^1^H] NOEs.

The relaxation experiments on the CGI-58 peptide V10-K43 revealed a clear separation of three distinct regions, namely flexible N- and C-terminal regions and a more rigid central region (Fig. 4). *T*_1_ times increase markedly between Trp^21^ and Ser^33^ (Fig. 4*A*), indicating reduced mobility. This is in agreement with the *T*_2_ values (Fig. 4*B*), which were less than 100 ms for residues between Trp^21^ and Ser^35^. *T*_2_ times less than 100 ms in the LD binding region correspond to motion dynamics of a large (>20 kDa) protein. This indicates that this region comprises the LD anchor and is embedded in the LD mimicking micelle. Moreover, the rapid dynamics observed for the terminal regions indicate that these regions move independently of the LD anchor. This is further substantiated with the ^15^N[^1^H] NOE experiment. Residues experiencing rapid internal motion flank a considerably more rigid core between Gly^20^ and Thr^32^ (Fig. 4*C*).

##### Paramagnetic Relaxation Enhancements Reveal the Immersion Depth of the Peptide Anchor

To elucidate the orientation of the CGI-58 peptide V10-K43 in DPC micelles and to determine the boundaries of the LD-binding motif, we recorded longitudinal relaxation experiments in the presence of various Gd(DTPA-BMA) concentrations. PREs collected in the presence of this highly water soluble and inert compound correlate with the insertion depths of peptide residues and can be converted to distance restraints during structure calculation (37, 38). Based on dynamic light scattering experiments, the hydrodynamic radius of the DPC micelles was calculated to be 30 Å in the presence of the CGI-58 peptide. For protons more than 28 Å from the micelle center, only lower distance restraints were generated. All protons calculated to be within 28 Å of the micelle center correspond to residues ranging from Trp^21^ to Trp^29^ and thus reaffirm the depth of immersion of the LD anchor motif (Table 1). The paramagnetic restraints are deposited with the accession number 5a4h at the Protein Data Bank.

**TABLE 1:** **PRE values measured for the peptide V10-K43** Distances to the center of the micelle were calculated with an estimated micelle radius of 30 Å. Only resonances corresponding to distances lower than 28 Å are shown. A list with all PRE values is deposited (PDB code 5A4H).

Residue	Atom name	PRE value	Distance to center
		*s*^−*1*^ *mm*^−*1*^	Å
Trp^21^	H	0.2338	27.7
Leu^22^	HA	0.0972	24.2
Thr^23^	H	0.1252	25.3
Thr^23^	HA	0.1417	25.8
Gly^24^	H	0.2258	27.6
Trp^25^	H	0.0911	23.9
Trp^25^	HE1	0.1168	25.0
Leu^26^	H	0.0737	22.9
Leu^26^	HB2	0.064	22.2
Thr^28^	H	0.153	26.2
Trp^29^	H	0.1224	25.2

##### Solution Structure of the N-terminal LD Anchor of CGI-58

**FIGURE 5.**
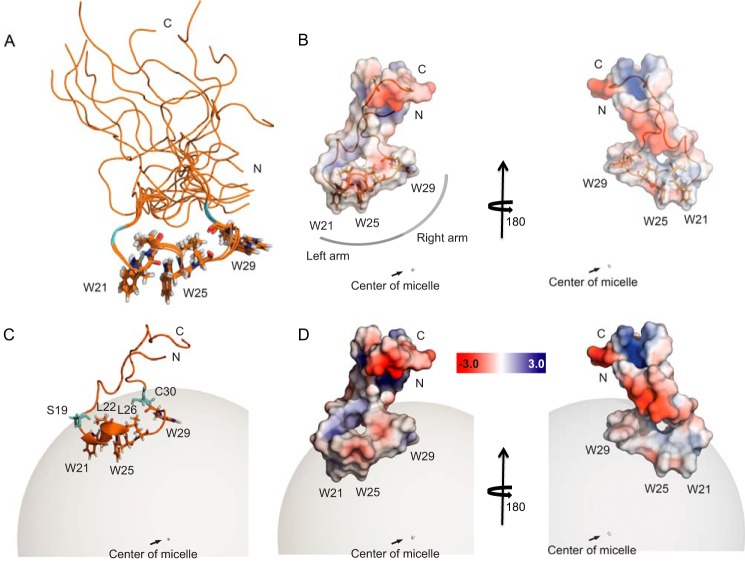
**Solution structure of the peptide V10-K43 in a DPC micelle.** *A,* overlay of 10 structures based on minimizing the backbone root mean square deviation between residues Gly^16^ and Thr^32^. Trp^21^, Trp^25^, Trp^29^, Leu^22^, and Leu^26^ are shown as sticks colored by atom, Ser^19^ and Cys^30^ are shown in *cyan. B,* the electrostatic potential on the solvent accessible surface of V10-K43 is shown as a semi-transparent surface. Hydrophobic residues anchoring the peptide in the LD mimicking DPC micelle form a hydrophobic arc along the interface with the micelle. They are depicted as *sticks* and colored by atom. *C,* V10-K43 is shown immersed into the DPC micelle. Ser^19^ and Cys^30^ delineate the interface between the micelle and the solvent. Three tryptophan and two leucine side chains fix the peptide in the micelle. *D,* representation of electrostatic potential on the surface of the peptide V10-K43 positioned in the DPC micelle. Solvent exposed stretches carry partial charges. The surface inside the micelle is increasingly hydrophobic as it approaches the micelle center.

**FIGURE 6.**
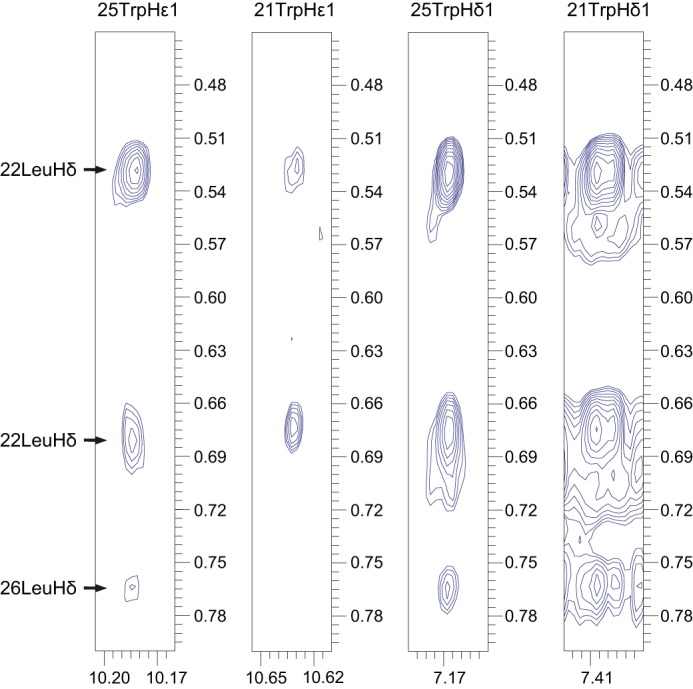
**Two-dimensional homonuclear ^1^H-^1^H NOESY strips of the peptide G18-E39.** Strong ^1^H-^1^H NOEs between indole rings of Trp^21^ and Trp^25^ and side chain δs of Leu^22^ and Leu^26^ confirm hydrophobic interactions between the Trp and Leu side chains.

To assess the differences in motion dynamics and establish the mode of binding of the peptide V10-K43, we calculated its solution structure immersed in DPC micelles. 533 NOE distance restraints were used for structure calculation, along with 36 backbone angular restraints (φ and ψ) and 66 PRE-derived distance restraints. The complete summary of quality statistics and experimental restraints is provided in Table 2. As expected, the backbone dihedral angles φ and ψ predominantly occupy coil regions of the Ramachandran plot (56). The flexible N- and C-terminal regions corresponding to residues Val^10^-Ala^15^ and Ser^33^-Lys^43^, respectively, are poorly defined due to the inherent dynamics in this region, as evidenced by the NMR relaxation experiments (Fig. 4). The central region ranging from Gly^16^ to Thr^32^ accounted for almost two-thirds (329 NOEs) of the NOE restraints and converged during structure calculation, revealing key aspects of LD binding. The predominantly hydrophobic and aromatic residues Ser^19^-Cys^30^ are immersed in the LD mimicking DPC micelles and constitute the LD anchor (Fig. 5). The left arm of this anchor comprises tryptophans 21 and 25 along with the leucines 22 and 26. These hydrophobic residues form a compact core along with a short helix between Gly^20^ and Gly^24^ (Fig. 5, *B* and *C*). A network of NOEs between tryptophan NHϵ-1 and NHδ-1 protons and the Leuδ-protons exemplifies these interactions (Fig. 6). Pro^27^ isolates Trp^29^ from the other tryptophans and together with Pro^31^ prevents the formation of a longer and more stable helix. The residue pairs Gly^18^/Ser^19^ and Cys^30^/Pro^31^ mark the interface between the DPC micelles and the solvent (Fig. 5*C*). A representation of the electrostatic potential on the solvent accessible surface of the peptide reveals the highly polar nature of the terminal segments and a predominantly hydrophobic LD anchor (Fig. 5, *B* and *D*).

**TABLE 2:** **Summary of quality statistics for an ensemble of 20 structures calculated with Cyana and list of experimental restraints**

Selected residues (SR)	16–32
Backbone root mean square deviations (Å)*^a^* for SR	0.61 ± 0.24
Heavy atoms root mean square deviations (Å)*^a^* for SR	0.77 ± 0.26
Backbone completeness*^a^*	96%
Side chain completeness*^a^*	74%
Ramachandran plot (%)*^b^* for SR	
Most favored regions	34.5
Additionally allowed regions	65.5
Generously allowed regions	0
Disallowed regions	0
Procheck φ - ψ (*Z*-score) for SR	−7.51
Procheck all (*Z*-score) for SR	−11.53
MolProbity Clashscore (*Z*-score) for SR	−3.06
Distance violations >0.5 Å	0
Angle violations >10°	3
All NOE restraints	533
Long (*li-jl* >5)	0
Short and medium (*li-jl* = 1 − 5)	285
Intra-residue	248
PRE distance restraints	66
Backbone angular restraints (φ and ψ)	36

*^a^* As defined by CING (43).

*^b^* Cumulative, calculated by PSVS using Procheck-NMR (75).

## Discussion

In this study, we describe the three-dimensional solution structure of the N-terminal LD anchor of CGI-58. Three anchor points (Trp^21^, Trp^25^, and Trp^29^) act synergistically to tether CGI-58 stably to LDs. The peptide sequence immersed within the LD comprises amino acids Ser^19^-Cys^30^. We demonstrate that a slightly longer CGI-58 sequence stretching from Ser^19^-Ser^35^ also recruits the otherwise cytosolic yellow fluorescent protein to LDs. However, this LD anchor lacks the ability to activate ATGL, indicating that other regions of CGI-58 are necessary for ATGL activation. The data presented here also corroborate earlier studies that LD binding of CGI-58 is a strict requirement for ATGL activation (15).

Single amino acid mutagenesis of any of the three tryptophans of the CGI-58 LD anchor and a variant lacking two tryptophan side chains (W21A/W25A) had no effect on CGI-58 LD co-localization. The ability of the CGI-58 variants to activate ATGL was reduced at most by one-third. In contrast, when we tested a W21A/W29A variant, the ability of CGI-58 to localize to LDs and to activate ATGL was completely abrogated. The unstructured nature of the LD anchor enables conformational flexibility and functional promiscuity. Therefore, it is conceivable that the CGI-58 LD anchor undergoes a conformational change upon CGI-58 binding to ATGL. The LD anchor might be necessary for the correct orientation of CGI-58 on LDs, which provides the platform for interaction with ATGL, or for positioning TGs favorably with respect to CGI-58 bound ATGL. Alternatively, CGI-58 might serve as mediator to transfer released FFAs from LD-bound ATGL to the water-soluble and cytosolic fatty acid-binding proteins, yet the LD anchor motif of CGI-58 is not required for binding to fatty acid-binding proteins or ATGL (15, 16). Again, the correct positioning of CGI-58 with respect to all interaction partners could be realized via the LD-anchor. Obviously, additional structural data of the involved binary and ternary complexes are required to learn more about this complex network.

The structures of membrane proteins have become a prominent and rapidly expanding field of structural biology, due to novel experimental and methodological breakthroughs. Structural studies of proteins acting at the water-lipid or membrane interface pose a tremendous experimental challenge. Consequently, the interaction of proteins with LDs remains largely uncharted territory. CGI-58 was initially demonstrated to bind to LDs and perilipin 1 simultaneously in adipocytes. Upon activation of lipolysis, CGI-58 dissociates from perilipin 1 and forms a LD bound complex with ATGL (57). Perilipin 1 independent binding of CGI-58 to LDs was demonstrated in *COS-7* cells (15, 58). Cell types that do not express perilipin 1 often express other members of the perilipin family (perilipins 2–5). Perilipin 5 interacts with CGI-58 and ATGL in a mutually exclusive manner (59) and the interactions of CGI-58 with perilipins 2 and 3 appear to be functionally less significant (60).

Only few structures of proteins that interact with LDs have been solved. Previously, Dunne and colleagues (61) showed the NMR structure of two CTP:phosphocholine cytidylyltransferase peptides in atomic detail on two overlapping 33- and 22-residue peptides, which span most of the amphipathic predominantly α-helical membrane-binding domain of rat CTP:phosphocholine cytidylyltransferase. The structural work on these peptides was performed in a membrane-binding context, yet *Drosophila* orthologues of CTP:phosphocholine cytidylyltransferase have been shown to localize to LDs as well (62, 63). Additionally, structures of a few soluble orthologues or domains of LD-binding proteins have been characterized (64, 65). The soluble C-terminal domain of the patatin family member TIP47 (perilipin 3) was solved nearly a decade ago (66), but the structure of the LD binding domain remains elusive. Intriguingly, the crystal structure of human monoacylglycerol lipase has been determined (67–69) and recently Nasr and colleagues (70) demonstrated that the cap region of human monoacylglycerol lipase interacts with nanodisc phospholipid bilayers, anchoring human monoacylglycerol lipase in a membrane-associated conformation. This leads to significantly increased *V*_max_ and decreased *K_m_* values for the substrates arachidonoyl 7-hydroxy-6-methoxy-4-methylcoumarin ester and 2-arachidonoylglycerol (70). In a recent study, a combination of deuterium exchange experiments and molecular dynamics simulations suggests the membrane interface to act as allosteric activator of phospholipases, inducing conformational changes from an inactive to an active conformation (71). Clearly, unveiling the structure-function relationship of proteins associated directly with LDs and membranes will provide important mechanistic insight into protein-lipid interactions essential for different physiological processes including membrane remodeling, lipid signaling, and intracellular lipolysis.

Peptides that anchor proteins to membranes are often transmembrane α-helices or α-helices, which orient perpendicularly to the membrane. The mechanism by which peptides anchor proteins to LDs remains unknown, but it is clear that a trans-LD α-helix is not feasible, as LDs assume diameters of 0.1 to 100 μm (72). The CGI-58 LD anchor observed here is not solely an α-helix that is perpendicular to the membrane. Within residues Ser^19^ to Leu^26^ of the LD-binding motif, Gly^20^ to Gly^24^ form a helix and the hydrophobic residues Trp^21^, Leu^22^, Trp^25^, and Leu^26^ establish a compact hydrophobic core, representing the left arm of the LD anchor. The conserved CGI-58 prolines Pro^27^ and Pro^31^ impede the formation of a longer continuous helix. The third tryptophan resides between these two prolines and establishes the structurally independent right arm of the LD anchor. As a consequence, the substitution of tryptophans Trp^21^ and Trp^25^ with alanines does not abrogate CGI-58 function, because the right anchor arm comprising Trp^29^ remains intact. On the other hand, replacement of Trp^21^ and Trp^29^ abrogates the function of CGI-58, because both anchor arms are affected by the substitutions. The importance of correct LD anchoring of CGI-58 is highlighted by conservation of the sequence of almost the entire LD anchor among vertebrates (3). The presence of two independently acting arms of the LD anchor could prevent defective anchoring as a result of malfunction in one LD anchor arm.

Selective inhibition of LD anchoring by CGI-58, or CGI-58 interactions with fatty acid-binding proteins, ATGL or perilipins, presents an opportunity for selective therapeutic targeting of lipid metabolism and peroxisome proliferator-activated receptor regulated gene expression. An essentially identical sequence to the LD anchor motif of CGI-58 can be found at the N terminus of a close relative of CGI-58, α/β hydrolase 4 (ABHD4) (55% sequence identity). Unlike CGI-58, ABHD4 is an active serine hydrolase and has been shown to hydrolyze *N*-acyl phosphatidylethanolamines (NAPEs) and lyso-*N*-acyl phosphatidylethanolamines (73). Moreover, ABHD4 was recently demonstrated to be a regulator of multiple classes of *N*-acyl phospholipids in the mammalian nervous system (74). From a drug design perspective the highly similar LD anchor of CGI-58 and ABHD4 can be both an opportunity and a limitation. Molecules that hinder CGI-58 LD anchoring may potentially affect ABHD4 activity as well. This would limit the specificity of an approach to target the LD anchor of CGI-58, but might also present a chance to trigger synergistic effects by influencing the activities of both proteins.

In summary, we show the structure of a LD anchor and describe the mechanism by which it binds to LDs using DPC micelles as LD mimics. The utilization of two independent LD binding arms reveals an intriguing strategy to protect CGI-58 against the loss of LD binding activity and highlights the importance of proper CGI-58 binding to LDs.

## Author Contributions

A. B., H. M. N., H. A., G. W., K. Z., and M. O. conceived and designed the experiments. A. B., H. M. N., H. A., C. J. P., H. L., and K. Z. performed the experiments. A. B., H. M. N., H. A., C. J. P., R. E. L., K. Z., and M. O. analyzed the data and A. B., H. M. N., R. E. L., G. W., R. Z., K. Z., and M. O. wrote the paper. All authors approved the final version of the manuscript.

## Footnotes

2 The abbreviations used are:

TG: triacylglycerol
CGI-58: comparative gene identification-58
ATGL: adipose triglyceride lipase
LD: lipid droplet
FFA: free fatty acid
DPC: dodecylphosphocholine
ABHD4: α/β hydrolase 4
TEV: tobacco etch virus
Gd(DTPA-BMA): gadolinium-diethylenetriamine pentaacetic acid-bismethylamide
PRE: paramagnetic relaxation enhancement
Ni-NTA: nickel-nitrilotriacetic acid.
